# Fabrication of Micro-Structured LED Diffusion Plate Using Efficient Micro Injection Molding and Micro-Ground Mold Core

**DOI:** 10.3390/polym12061307

**Published:** 2020-06-08

**Authors:** Yanjun Lu, Wang Luo, Xiaoyu Wu, Bin Xu, Chunjin Wang, Jiajun Li, Liejun Li

**Affiliations:** 1 Guangdong Provincial Key Laboratory of Micro/Nano Optomechatronics Engineering, College of Mechatronics and Control Engineering, Shenzhen University, Shenzhen 518060, China; 1810293039@email.szu.edu.cn (W.L.); wuxy@szu.edu.cn (X.W.); lijiajunszu@163.com (J.L.); 2 Partner State Key Laboratory of Ultra-precision Machining Technology, Department of Industrial and Systems Engineering, The Hong Kong Polytechnic University, Hong Kong; chunjin.wang@polyu.edu.hk; 3 Guangdong Key Laboratory for Processing and Forming of Advanced Metallic Materials, School of Mechanical and Automotive Engineering, South China University of Technology, Guangzhou 510640, China

**Keywords:** micro-structure, diffusion plate, micro injection molding, grinding

## Abstract

In this paper, a new style of micro-structured LED (light-emitting diode) diffusion plate was developed using a highly efficient and precise hybrid processing method combined with micro injection molding and micro-grinding technology to realize mass production and low-cost manufacturing of LED lamps with excellent lighting performance. Firstly, the micro-structured mold core with controllable shape accuracy and surface quality was machined by the precision trued V-tip grinding wheel. Then, the micro-structured LED diffusion plate was rapidly fabricated by the micro injection molding technology. Finally, the influences of micro injection molding process parameters on the illumination of the micro-structured diffusion plate were investigated. The simulated optical results show that the illumination of the micro-structured diffusion plate can achieve a maximum value when the V-groove depth and V-groove angle are designed to be 300 μm and 60°, respectively. The experimental results indicate that the developed micro-structured diffusion plate may improve the illumination by about 40.82% compared with the traditional diffusion plate. The prediction accuracy of the designed light efficiency simulation method was about 90.33%.

## 1. Introduction

LED lamps are widely used in the lighting field due to their advantages of being energy efficient and environmentally friendly and producing a uniform and soft light. [1,2]. To further improve the luminous efficiency of LED lamps and reduce their production and manufacturing costs, many researchers have focused on the fabrication of the micro-array structure on the diffusion lampshade of LED lamps [3,4], which can reduce the diffusion and scattering of light to improve the utilization rate of light energy.

At present, the fabrication process of the micro-structured diffusion plate mainly includes silk-screen printing, hot pressing, and micro injection molding. Silk-screen printing can be used to directly fabricate a diffusion plate and has the advantages of low manufacturing cost and high production efficiency. It can print a uniform macroscopic dot structure, resulting in uniform light. However, the silk-screen printing process was complex, and the generated ink volatilization polluted the environment [5]. Previous studies have shown that the micro-structure was first machined on the surface of a mold and then was replicated to the polymer workpiece surface to rapidly fabricate the micro-structured polymer diffusion plate by hot pressing or micro injection molding technologies [6,7] to realize the mass production and manufacture of micro-structured diffusion plates. Due to its simple manufacturing process and low production cost, micro injection molding is suitable for the mass production and manufacture of micro-structured polymers. Besides, it is almost free from the limitation of plastic parts’ geometry. Therefore, the micro injection molding technology has become the main forming process for micro-structured polymer products [8,9]. For example, the relationship between mold micro-manufacturing and the micro injection molding process was studied to optimize the demolding phase [10]. The hybrid method combining optical micro metrology and injection molding process monitoring was proposed to verify the quality of 3D micro-molded components [11]. The effects of milling strategies and cutting parameters on the mold core were investigated to reveal the relationship between mold topography and the ejection force in micro injection molding [12]. The relationship between different surface topography parameters and the ejection force in micro injection molding was analyzed to describe the friction behavior at the polymer–tool interface [13].

The forming quality of the micro-structured polymer products mainly depends on the machining quality and shape accuracy of the micro-structured mold core. The common micro-machining technologies of micro-structured mold core include electrochemical etching [14], laser processing [15], electrical discharge machining (EDM) [16], and mechanical micro-cutting [17,18], ect. Although chemical etching can ensure the shape accuracy of a nanoscale structure [14], it was impossible to efficiently and precisely machine a 3D micro-structure at the micron scale. In order to greatly improve the mold etching precision, the production cost would be greatly increased. Besides, the chemical etching solution would also pollute the environment. High-efficiency laser processing is environmentally friendly [15], but the equipment cost is considerably expensive and it is very difficult to control the shape accuracy of the 3D micro-structure. Although the micro-EDM technology can produce micro-structures of less than 100 microns in size [16], it was limited by conductive mold materials. Unfortunately, the surface quality of the 3D micro-structure was not satisfactory, and the EDM oil polluted the environment. A micro-structure with high surface quality can be processed by the mechanical diamond cutting [17,18], but this method was limited to soft metal materials with low hardness. Besides, the depth-to-width ratio of the machined micro-structure was also relatively low. Therefore, under the premise of environmental protection and high efficiency, it is very difficult to process a micro-structured mold with high shape accuracy and surface quality at the micron scale. The previously developed micro-grinding technology may be used to machine high-quality microarray structures with controllable shape accuracy on the surface of hard and brittle mold materials [19,20]. Moreover, the environmentally friendly and pollution-free processing method has a low production cost and was a simple operation. At present, there are no relevant research reports on the fabrication of micro-structured LED diffusion plates using micro-grinding and micro injection molding. Therefore, the precision trued grinding wheel tool is first proposed to machine a micro-array structure with high surface quality and shape accuracy on the surface of mold core, and then the 3D micro-structure derived from mold core surface will be duplicated to the acrylic polymer lampshade by micro injection molding to rapidly fabricate a high-quality micro-structured LED diffusion plate, leading to an improvement in light efficiency of LED lamp.

In this paper, the hybrid processing method combining with micro-grinding and micro injection molding is proposed to efficiently fabricate a micro-structured LED diffusion plate with high surface quality and forming accuracy. Firstly, the optical simulation method was designed to simulate the distribution of light intensity on the micro-structured diffusion plate to obtain optimal micro-structure size parameters. Then, according to the micro-structure parameters obtained by simulation, the V-shaped groove array structure with high shape accuracy was fabricated by precision micro-grinding technology on the surface of the mold steel. Finally, the V-grooved array structure on the surface of the mold core was rapidly replicated to the surface of the acrylic polymer diffusion plate by the micro injection molding process to efficiently produce a new type of micro-structured LED diffusion plate. The influences of micro injection molding process parameters on the light intensity of the LED diffusion plate were investigated to obtain the optimal injection process parameters.

## 2. The Optical Design and Light Efficiency Simulation of the Micro-structured LED Diffusion Plate

LED diffusion plate material is usually polymethyl methacrylate (PMMA), which has excellent optical properties such as high white light penetration, high refractive index, and high light transmittance. Its Mohs hardness and refractive indexes are 95 kg/cm^2^ and 1.49, respectively. According to previous investigations [21,22], the optical performance can be effectively improved by fabricating the micro-structures on the surface of the LED diffusion plate. In order to efficiently design the optical structure for the LED diffusion plate, the TracePro software was employed to simulate the luminous flux and illuminance of traditional and micro-structured LED diffusion plate surfaces to evaluate their light efficiencies.

**Figure 1:**
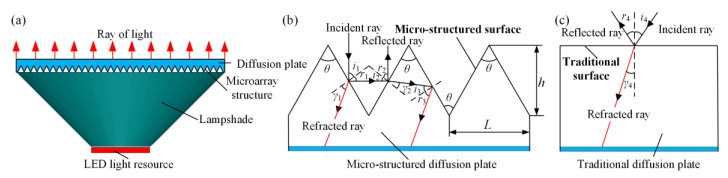
(**a**) Schematic diagram of the LED lamp. (**b**) Optical travel principle of the microstructured diffusion plate. (**c**) Optical travel principle of the traditional diffusion plate.

According to the light-emitting principle of the diffusion plate, it is very feasible for the micro-structure to be designed as a V-shaped groove. A common LED lamp mainly contains an LED light source, a lampshade, and a diffusion plate (see Figure 1a). The ray of light emitted by the light source is refracted from the surface as it travels through the diffusion plate. The outside diameter and thickness of the diffusion plate sample were 50 and 1.3 mm, respectively. The LED light source was a lamp bead with a rated power of 3 W and package size of 10 mm in diameter and 1 mm in thickness. The distance between the light source and the diffusion plate was 18 mm, and the simulated light ray was visible light with a wavelength of 400–700 nm. The wavelength of the simulated light ray was set as 450 nm, and the number of experimental light rays was 10,000. As seen from Figure 1a, the side of the diffusion plate with regular micro-array structure was regarded as the incident surface, and the backside was set as the light-emitting surface. The V-groove angle, V-groove depth, and V-groove width of the micro-array structure on the surface of diffusion plate sample were labeled as *θ*, *h*, and *L*, respectively (Figure 1b). Refraction and reflection would be produced as the incident ray travels through the diffusion plate. The incident ray, reflected ray, and refracted ray were expressed as *i*, *r*, and *γ*, respectively. The optical travel principles of the micro-structured and traditional diffusion plates are shown in Figure 1b,c. As observed from Figure 1b,c, in qualitative terms, compared with the traditional surface, the micro-structured surface may produce a more refracted and reflected ray to improve the light efficiency of the LED lamp. The simulated luminous flux and illuminance derived from the diffusion plate were recorded to evaluate the light efficiency.

In order to obtain the optimal microgroove structure sizes, the V-groove depth *h* of 100–400 µm and V-groove angle *θ* of 60°–150° were designed and changed to calculate the theoretical illuminance *E* (see Table 1). Table 1 shows the simulated illuminances of the LED diffusion plate under different micro-structure parameters.

**Figure 2:**
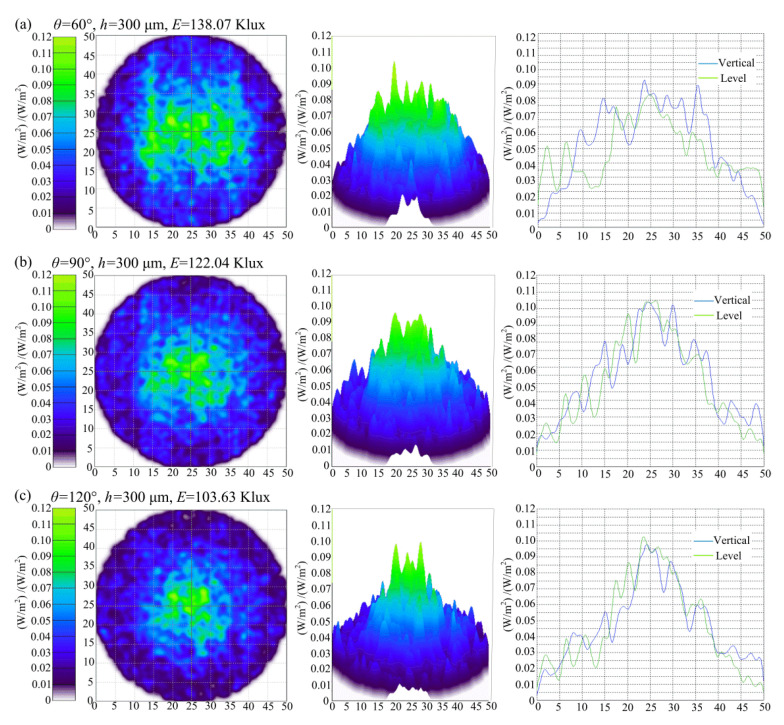
The simulated irradiances and illuminances of micro-structured LED diffusion plates depending on the designed V-groove angle *θ*: (**a**) *θ* = 60°; (**b**) *θ* = 90°; (**c**) *θ* = 120°.

Figure 2 shows the influence of the designed V-groove angle *θ* on the irradiance and illuminance of the micro-structured LED diffusion plate. A brighter color indicates a higher illumination intensity. The diameter of the diffusion plate was 50 mm, and the light source was located directly below the diffusion plate. As seen from the irradiance and illuminance image of the diffusion plate, areas of lower light intensity of the area were further from the center of light source and resulted in darker images. It can also be seen that the highest light intensity was located in the center area. When the V-groove depth *h* remained consistent, the light intensity decreased with the increase of V-groove angle *θ*. When the V-groove angle was 60°, the light intensity was the highest, at this time, the highest illuminance *E* reached 138.07 Klux.

**Figure 3:**
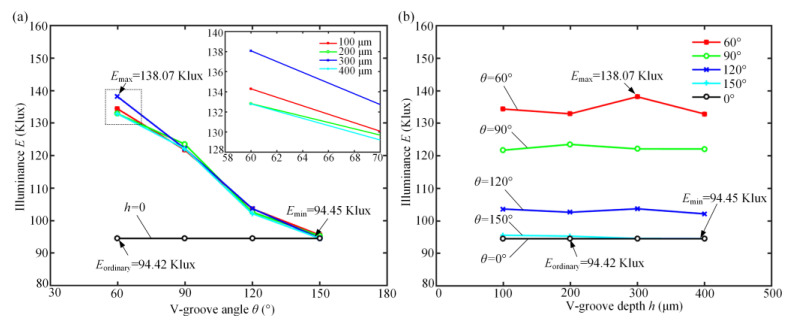
The simulated illuminance *E* as influenced by the designed V-groove structure parameters: (**a**) V-groove angle *θ*; (**b**) V-groove depth *h*.

Figure 3 shows the influence of the designed V-groove structure parameters on simulated illuminances. It is shown that the influence of the V-groove angle on luminous flux was greater than the influence of V-groove depth. Within the V-groove angle range of 60 to 150°, a smaller V-groove angle resulted in a greater illumination intensity (see Figure 3a). It can be observed that when the V-groove depth was between 100 and 400 µm, there was little change in illumination intensity as the V-groove depth increased (see Figure 3b). The illumination intensity was significantly improved by designing a specific V-groove structure on the surface of the diffusion plate. Compared with the simulated illumination *E*_traditional_ of 94.42 Klux for the traditional diffusion plate, the maximum illumination *E*_max_ of 138.07 Klux for the micro-structured diffusion plate may be theoretically enhanced by about 46.23%. Greater groove depths cause the grinding wheel to wear faster, necessitating frequent dressing and truing for the grinding wheel. Therefore, comprehensively considering the actual processing conditions and simulation results, the optimal micro-groove parameters of a V-groove depth of 300 μm and V-groove angle of 60° were chosen in the experiments.

## 3. Experimental Details

The surface quality and shape accuracy of V-groove structure machining of the mold core surface mainly depend on the dressing and truing accuracy of CBN (Cubic boron nitride) grinding wheel V-tip. Therefore, before the grinding experiment, the previously developed precision dressing and truing technology [23,24] was first implemented to produce a V-tip grinding wheel with a V-tip angle of 60°. Then, the trued #600 resin-bonded CBN grinding wheel was employed to machine regular V-grooved array structures with the V-groove depth of 300 μm on the surface of the mold core. Next, the micro-structured LED diffusion plate was fabricated by micro injection molding technology based on a micro-ground mold core. Finally, the light efficiencies of traditional and micro-structured LED diffusion plates were comparatively tested.

### 3.1. Precision Truing of the V-Tip CBN Grinding Wheel

**Figure 4:**
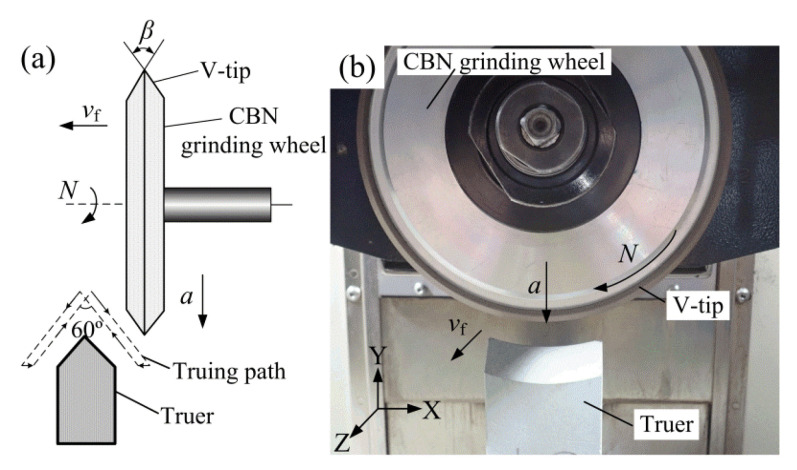
The precision truing of the V-tip CBN grinding wheel: (**a**) V-tip truing principle; (**b**) truing photo.

Figure 4 shows the V-tip truing principle and photo of the CBN (Cubic boron nitride) grinding wheel. The machine tool is a three-axis precision CNC plane grinder (SMART-B818 III, Chevalier, Taiwan, China) with a minimum feed of 1 μm. Firstly, a macroscopic V-shaped tip grinding wheel was rapidly formed through rough truing along a V-shape interpolation truing path using the #80 coarse oilstone truer (green SiC) (see Figure 4a). Then, the #600 oilstone was used for fine truing to obtain a V-tip with high shape accuracy and sharp micro-grain cutting edges. The theoretical V-tip truing angle was set as 60°. The V-tip angle of the trued grinding wheel was labeled as *β*.

### 3.2. Precision Grinding of the Micro-structured Mold Core

**Figure 5:**
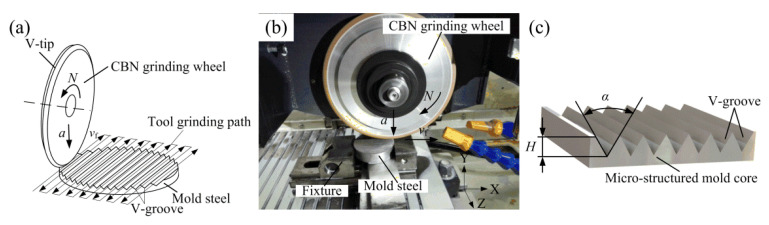
The precision grinding of the micro-structured mold core: (**a**) grinding principle and path; (**b**) experimental photo; (**c**) schematic diagram of the V-groove structure.

In this study, S136H mold steel with the hardness of 30–35 HRC was chosen as the mold core material due to its excellent wear resistance and machinability, and it was polished to a mirror-like surface finish before micro-structure grinding. Figure 5 shows the V-groove array structure grinding principle and an experimental photo of the mold core. The precision trued V-tip CBN grinding wheel was driven by the CNC system to grind the mold steel installed on the horizontal worktable by the fixture (see Figure 5a). The V-groove was machined on the surface of mold steel by replicating the V-tip shape of the CBN grinding wheel along the set horizontal reciprocating cutting path. When a V-groove was machined, the grinding wheel was moved at a set pace along the *Z*-axis direction to conduct the machining of the second V-groove. Finally, the micro-structured mold core with regular and controllable V-grooved array structures was fabricated (see Figure 5b). According to previously determined fundamental experimental parameters, to ensure the grinding quality and production efficiency, the precision grinding conditions of the micro-structured mold core, including wheel speed *N*, depth of cut *a*, and feed speed *v*_f_, were chosen and are shown in Table 2. The V-groove angle *θ* and V-groove depth *h* were set as 60° and 300 μm, respectively. The V-groove angle and V-groove depth of the ground micro-structured mold core surface are expressed as α and H, respectively (see Figure 5c).

### 3.3. Micro injection Molding of the Micro-structured LED Diffusion Plate

**Figure 6:**
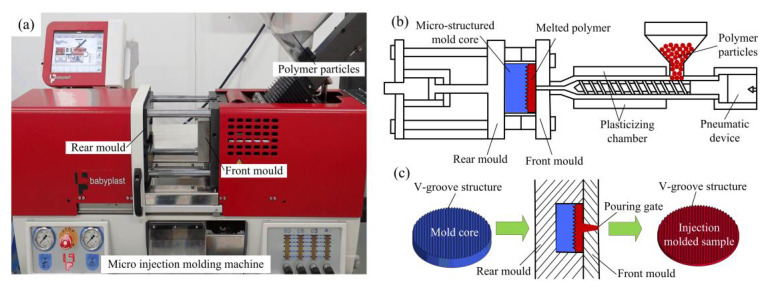
The micro injection molding of the micro-structured diffusion plate: (**a**) experimental photo; (**b**) schematic diagram of micro injection molding; (**c**) molding principle of the injection molded sample.

The efficient micro injection molding technology was developed to replicate the V-grooved array structure of the mold core surface to the PMMA surface to precisely form the micro-structured diffusion plate sample through a micro injection molding machine (6/10P, Babyplast, Hospitalet Llobregat, Spain). Figure 6 shows a micro injection molding photo and the molding principle of a micro-structured LED diffusion plate. Firstly, the ground V-groove array structured mold core was installed on the rear mold, and the PMMA (8817, Degussa, Frankfurt, Germany) polymer particles were put into the hopper (see Figure 6a). Then the polymer particles were driven by a pneumatic device into a plasticizing chamber. Next, the plasticized and melted polymer flowed into the front mold through the pouring gate. Finally, the close-fitting rear mold and front mold were separated after cooling and holding pressure (see Figure 6b). Thus, the V-groove structure of the mold core surface was copied on the polymer workpiece surface to fabricate the V-shaped injection molded sample (see Figure 6c).

The micro-forming quality of the injection molded diffusion plate sample can affect the light efficiency of the LED lamp. Therefore, the optimal micro injection molding process parameters needed to be determined to achieve the maximum light efficiency of the micro-structured diffusion plate. To do this, the influences of the micro injection molding process parameters on the forming accuracy and surface quality of the micro-structured polymer sample needed to be studied. Under general injection molding conditions, the ideal mold temperature for PMMA injection is about 40–70 °C. According to previous experimental results [19], the molding quality of a micro-structured polymer was satisfactory at room temperature in this experiment. Therefore, the mold temperature was not considered as an injection molding process parameter in this work. The common micro injection molding process parameters, including melt temperature, injection speed, injection pressure, holding pressure, and holding time, were chosen and experimental designed in this work as listed in Table 3. Under each process parameter, ten micro injection molded samples were captured for testing to reduce the experimental error.

### 3.4. Light Efficiency Testing of the Micro-structured LED Diffusion Plate

**Figure 7:**
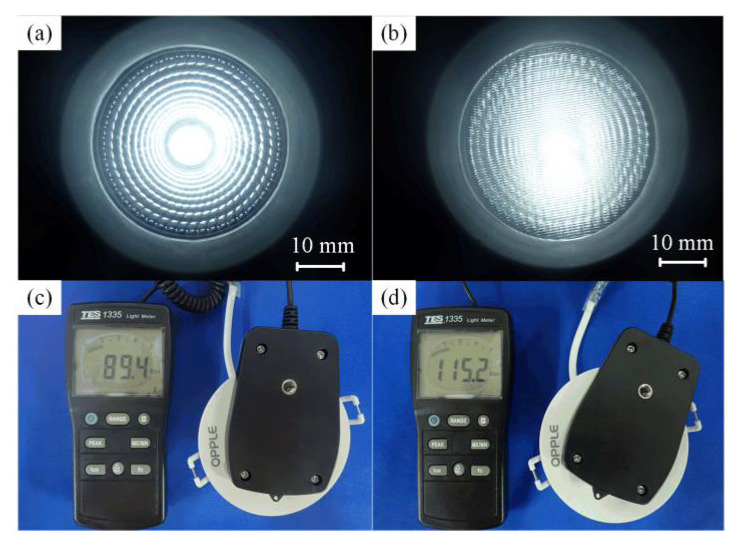
The light efficiency testing photos of the LED diffusion plates: (**a**) traditional diffusion plate; (**b**) micro-structured diffusion plate; (**c**) Light intensity of traditional diffusion plate; (**d**) Light intensity of micro-structured diffusion plate.

In order to obtain maximum light efficiency, the digital illuminometer (1335, TES, Taiwan, China) with the measurement resolution of 0.01–0.1 Klux was employed to test the illuminance of micro-structured LED diffusion plates under different micro injection molding process parameters. The average value of five measured illumination values was regarded as the average illumination *E*. The light source was a commercial LED embedded lamp (LEH0103010, OPPLE, Shanghai, China) with the rated power of 3 W, rated voltage of 220 V, and an rated current of 0.025 A. Figure 7 shows the light efficiency testing photos of the traditional and micro-structured LED diffusion plates. Here, the injection molding process parameters of melt temperature, injection speed, injection pressure, holding pressure, and holding time were 245 °C, 40 mm/s, 8 MPa, 5 MPa, and 2.5 s, respectively. It is shown that the illuminance values for the traditional and micro-structured LED diffusion plates were 89.4 and 115.2 Klux, respectively. This indicates that the illuminance of the micro-structured diffusion plate may be improved by about 28.8% compared with the traditional one.

To evaluate the machining quality of the micro-ground mold core and the micro-forming quality of the injection molded LED diffusion plate sample, a probe stepper (D-300, KLA-Tencor, California, USA) was adopted to capture the section profiles of the micro-structured mold core and polymer workpieces. A 3D laser scanning microscope (VK-X250K, Keyence, Osaka, Japan) was used to test the 3D topographies of the micro-structured mold core and micro-formed polymer samples. High-resolution scanning electron microscopy (SEM, FEI Quanta 450 FEG and Apreo S, FEI Company, Hillsboro, USA) was employed to detect the surface topographies of the micro-structured mold core and injection-molded polymer samples. Through the measured section profile curves, the V-groove structure parameters of the micro-ground mold core and micro-formed diffusion plate sample can be obtained. The V-groove structure parameters of the micro-structured mold core are described as width V-groove angle *α*, V-groove depth *H*, and V-tip radius *R*. The V-groove structure parameters of the micro-structured diffusion plate sample are described as V-groove angle *θ*, V-groove depth *h*, and V-tip radius *r*. The presented result was the average value of three measurements.

## 4. Results and Discussion

### 4.1. Truing Accuracy of the Grinding Wheel V-Tip

**Figure 8:**
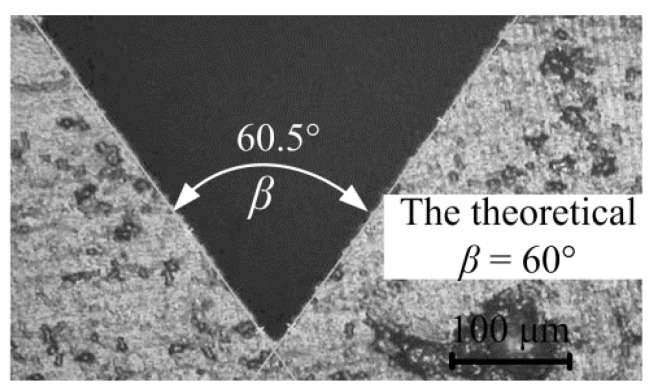
The V-tip section shape of trued grinding wheel.

Generally, the grinding wheel tip will slightly wear in the grinding process, but the tip angle of the grinding wheel will not change at the macro-level. To reduce wear and improve shape accuracy of the V-groove machining, the grinding wheel was trued at regular intervals to ensure the processing quality and efficiency. Figure 8 shows the V-tip section shape of #600 CBN grinding wheel after precision V-tip truing. The V-tip section shape of the trued grinding wheel can be obtained by cutting and replicating a V-groove on the surface of the carbon graphite plate [25]. It is shown that the trued V-tip angle *β* was about 60.5°. The V-tip truing angle error was only 0.5° compared with the theoretical V-tip angle of 60°. Therefore, the previously developed grinding wheel V-tip truing technology [19] can ensure the shape accuracy of micro-structure grinding machining, facilitating micro-forming accuracy of the micro-structured polymer diffusion plate in the micro injection molding process.

### 4.2. Photos and Surface Topographies of the Micro-ground Mold Core and the Injection Molded Micro-structured Diffusion Plate

**Figure 9:**
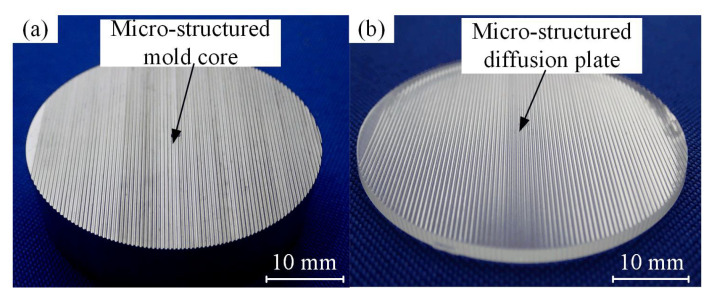
Photographs of the micro-structured mold core and diffusion plate: (**a**) micro-ground mold core; (**b**) injection molded diffusion plate sample.

Figure 9 shows the photographs of the micro-ground mold core and the injection molded micro-structured LED diffusion plate. It is shown that the regular and smooth V-groove array structures without spacing were machined on the surface of mold steel mold core by micro-grinding technology (see Figure 9a). After micro injection molding, the inverted V-shaped groove array structures were fabricated on the surface of PMMA polymer to form the micro-structured diffusion plate by replicating the micro-structure characteristic derived from the mold core. The measured surface roughness *R*_a_ values of the V-grooved surface for the mold core and diffusion plate were 0.42 μm and 0.148 μm, respectively. It can be concluded that the surface quality of the injection molded micro-structured diffusion plate was significantly higher than that of the mold core. Under the action of injection pressure, this is because the molten polymer produces contraction after a period of compression and cooling in the process of micro injection molding, leading to a dense and smooth surface. Therefore, the proposed precision grinding and micro injection molding technologies have the potential for the mass production and manufacturing of micro-structured LED diffusion plates.

**Figure 10:**
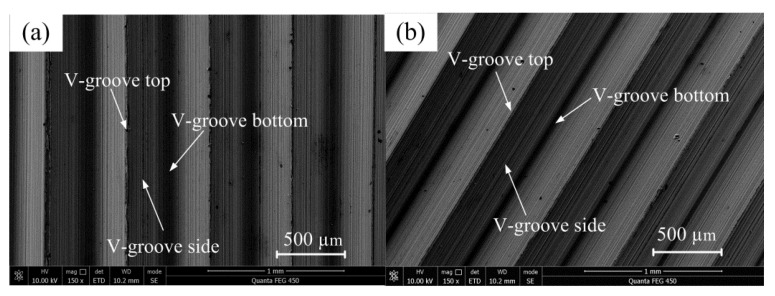
SEM photos of the micro-structured mold core under different orientations: (**a**) vertical; (**b**) inclined.

Figure 10 shows the SEM photos of the micro-structured mold core under vertical and inclined orientations. By observing the surface topographies of the mold core along vertical and inclined orientations, it is found that the microscopic V-groove structures were considerably regular and integral. Specifically, the V-groove top had hardly any damage, and the contour of the V-groove bottom was clearly identifiable. There was an existing arc radius at the V-groove bottom due to the inevitable V-tip truing error and micro-grinding machining error. Therefore, the V-groove array structure with regular and controllable shape accuracy can be machined on the surface of mold steel mold core through precision micro-grinding technology.

**Figure 11:**
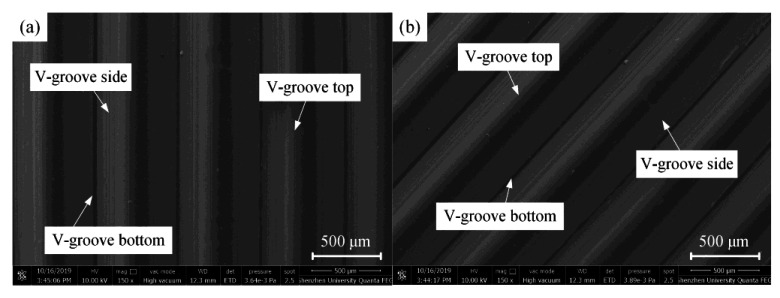
SEM photos of the injection molded micro-structured diffusion plate sample under different orientations: (**a**) vertical; (**b**) inclined.

Due to a large number of diffusion plate samples, it is not necessary to show all the SEM photos. Therefore, the diffusion plate sample prepared under one group of process parameters was selected for SEM image characterization. Here, the injection molding process parameters of melt temperature, injection speed, injection pressure, holding pressure, and holding time were 250 °C, 40 mm/s, 8.0 MPa, 5.0 MPa, and 2.5 s, respectively. Figure 11 shows the SEM photos of the injection molded micro-structured diffusion plate sample under vertical and inclined orientations. It can be seen that the injection molded polymer sample surface was rather smooth, and the micro-formed V-groove array structure was also highly regular and integral. Moreover, there was no burr or damage at the top and bottom of the V-groove. This indicates that a micro-structured polymer sample with high injection molding quality can be obtained by using the proposed micro injection molding technology along with a micro-structured mold core with high shape accuracy. Compared with the SEM morphology of the mold core shown in Figure 10, it is found that the V-groove side surface of the micro-structured polymer diffusion plate was smoother. The melt viscosity of PMMA is mainly governed by pressure, so the injection pressure was increased to reduce the viscosity and increase the fluidity, giving the melted polymer good fluidity and contractility and facilitating the fabrication of a smooth micro-structured diffusion plate.

### 4.3. The Section Profiles of the Micro-structured Mold Core and Diffusion Plate

**Figure 12:**
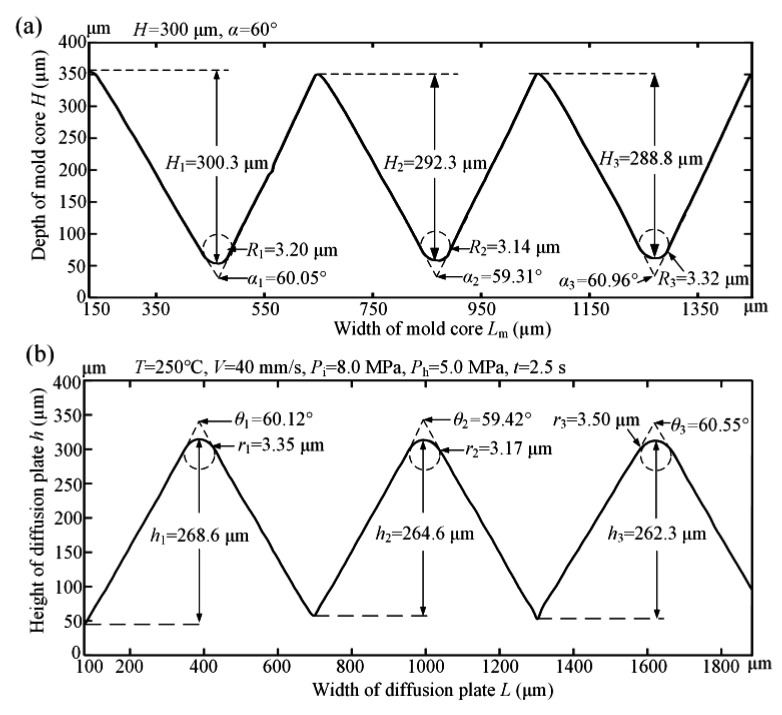
The section profile curves of the micro-structured mold core and diffusion plate sample: (**a**) mold core; (**b**) diffusion plate sample.

The probe stepper was employed to capture the section profile curves of the micro-structured mold core and diffusion plate sample, as shown in Figure 12. As seen from Figure 12a, the measured average V-groove depth *H* and V-groove angle *α* of micro-ground mold core were 293.8 μm and 60.11°, respectively. Compared with the theoretical designed V-groove depth of 300 μm and V-groove angle of 60°, the micro-machining errors of V-groove depth and V-groove angle were only 6.2 μm and 0.57°, respectively. The average V-tip radius R of the ground V-groove structure was 3.22 μm. By comparing the theoretical with the actual V-groove profile curves of the mold cores, the shape error of micro-structure machining could be calculated. The shape error of the micro-structured mold core is mainly concentrated at the V-groove top due to the ineradicable V-tip arc radius. The absolute difference between the peak value and valley value of the V-groove profile curve was defined as the shape accuracy [19,25]. Thus, the shape accuracy of the micro-ground mold core was calculated to be 9.2 μm. The results indicate that a micro-structured mold core with high shape accuracy can be manufactured by micro-grinding technology, which was beneficial to produce an injection molded diffusion plate with high forming quality. The depth, angle, and tip radius of the three V-groove profiles on the front of the mold core and injection molded part was measured to calculate the average value to evaluate the shape accuracy. Figure 12b shows the profile curve of the micro-structured injection-molded diffusion plate sample. At this point, the micro injection molding process parameters, namely, the melt temperature, injection speed, injection pressure, holding pressure, and holding time were 250 °C, 40 mm/s, 8.0 MPa, 5.0 MPa, and 2.5 s, respectively. As shown in Figure 12b, the measured average V-groove depth *h* and V-groove angle *θ* of the injection-molded micro-structured diffusion plate sample were 265.2 μm and 60.03°, respectively. Compared with the V-groove depth and V-groove angle of the micro-ground mold core shown in Figure 12a, the micro-forming errors of V-groove depth and V-groove angle of the micro-structured diffusion plate were 28.6 μm and 0.08°, respectively. The average V-tip radius *r* of the micro-formed diffusion plate was 3.34 μm. As a result, compared with the V-tip radius of the mold core, the micro-forming error of the V-tip radius for the micro-structured diffusion plate was calculated to be 0.12 μm. Therefore, the developed micro injection molding using a micro-ground mold core can precisely fabricate a micro-structured diffusion plate with high molding accuracy. The replication rate was defined as the ratio of the micro-structural depth of the micro injection molded part to the micro-structural depth of the mold core [26,27]. Therefore, according to the average V-groove depth of the micro-structured mold core and diffusion plate sample, the replication rate of micro injection molded polymer sample was computed to be about 90.26%. The average standard deviations of the V-groove depth, V-tip angle, and V-tip radius of mold core were 5.89 μm, 0.83 μm, and 0.09 μm, respectively. The average standard deviations of the V-groove depth, V-tip angle, and V-tip radius of the diffusion plate were 2.82 μm, 0.57 μm, and 0.17 μm, respectively. It can be seen that the standard deviations for the micro-structured diffusion plate were relatively less than those of mold core.

### 4.4. Light Efficiency Analysis of the Micro-structured LED Diffusion Plate

**Figure 13:**
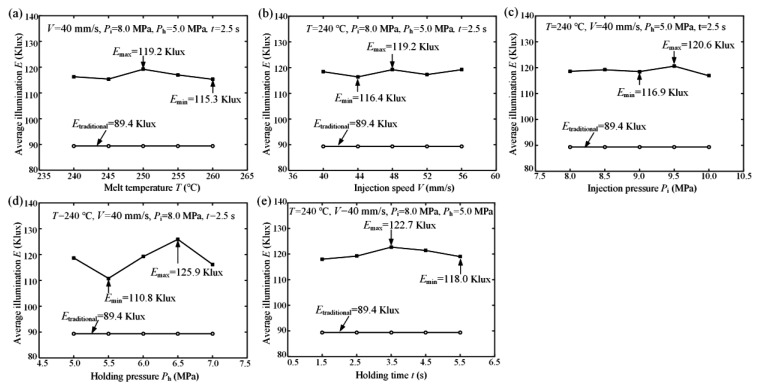
The average illuminations *E* of LED diffusion plates depending on micro injection molding process parameters: (**a**) melt temperature *T*; (**b**) injection speed *V*; (**c**) injection pressure *P*_i_; (**d**) holding pressure *P*_h_; (**e**) holding time *t*.

In order to study the influences of melt temperature, injection speed, injection pressure, holding pressure, and holding time on the light efficiency of the micro-structured diffusion plate to determine the optimal micro injection molding process conditions, the relationships between average illumination *E* of the micro-structured LED diffusion plate and different micro injection molding process parameters were determined and are described in Figure 13. It is shown that the measured average illumination *E*_traditional_ of the traditional diffusion plate was 89.4 Klux. As observed in Figure 13, the average illumination *E* values ranged from 115.3 to 119.2 Klux, 116.4 to 119.2 Klux, 116.9 to 120.6 Klux, and 118.0 to 122.7 Klux with the change of melt temperature, injection speed, injection pressure, and holding time, respectively (see Figure 13a–c,e). There was a variation of about 13% in optical performance. This was mainly attributed to the replication of the V-groove geometry controlled by micro injection molding process parameters. Because the V-groove has a tip arc, the differences in the replication of the groove geometry eventually lead to differences in optical performance. There were no significant changes in the average illumination when the melt temperature, injection speed, injection pressure, or holding time increased. The average illumination *E* ranged from 110.8 to 125.9 Klux with the change of holding pressure (see Figure 13d). This indicates that the average illumination had an obvious variation with the variation of holding pressure. The average illumination *E* of the injection molded micro-structured diffusion plate reached a maximum value of 125.9 Klux where the optimal micro injection molding process parameters, namely the melt temperature, injection speed, injection pressure, holding pressure, and holding time, were 240 °C, 40 mm/s, 8.0 MPa, 6.5 MPa, and 2.5 s, respectively. The experimental results show that the holding pressure had the greatest effect on the light efficiency of the micro-structured diffusion plate, while the other micro injection molding process parameters had little effect. Because the illumination of the traditional diffusion plate was 89.4 Klux, the maximum illumination of the micro-structured diffusion plate may be improved by about 40.82%. For the micro-structured diffusion plate, the experimentally measured illumination ranged from 110.8 to 125.9 Klux, which was very close to the simulated theoretical illumination of 138.07 Klux. Therefore, it was feasible to design the micro-structured LED diffusion plate through the light efficiency simulation method.

**Figure 14:**
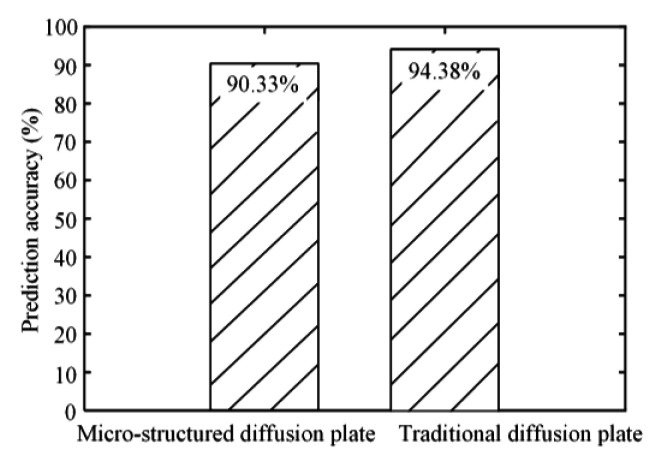
The prediction accuracies of traditional and micro-structured diffusion plates.

Figure 14 shows the prediction accuracies of traditional and micro-structured diffusion plates. When the V-groove depth *h* and V-groove angle *θ* were designed as 300 μm and 60°, respectively, the illumination value of the micro-structured diffusion plate determined by the designed optical simulation method was 138.07 Klux. The experimental maximum illumination value of the injection molded micro-structured diffusion plate was 125.9 Klux. Therefore, the prediction accuracy for the micro-structured diffusion plate was about 90.33%. The simulated and measured experimental illumination values of the traditional diffusion plate were 94.42 and 89.4 Klux, respectively. Therefore, the prediction accuracy for the traditional diffusion plate was about 94.38%. As a result, the designed light efficiency simulation method with high prediction accuracy may be used to conduct the structural design of a micro-structured LED diffusion plate. The proposed efficient micro injection molding technology based on a micro-ground mold core can realize the mass production and manufacturing of micro-structured LED diffusion plate products with high forming accuracy.

## 5. Conclusions

In this paper, the light efficiency simulation method was designed to obtain the optimal micro-structure parameters of a micro-structured LED diffusion plate. High-precision micro-grinding technology was proposed for the fabrication of regular and controllable micro-grooved array structures on the surface of a mold core. Efficient micro injection molding was employed to rapidly fabricate a micro-structured diffusion plate with high forming accuracy. The proposed hybrid fabrication method can realize mass production and manufacturing of micro-structured LED diffusion plates due to its low manufacture cost. The main results are summarized as follows:

1. Through the light efficiency simulation method, the optimal micro-groove parameters, namely V-groove depth and V-groove angle, were designed to be 300 μm and 60°, respectively.
2. The machining errors of the V-groove depth and V-groove angle for the micro-ground mold core were 6.2 μm and 0.57°, respectively. The micro-forming errors of the V-groove angle and V-tip radius for the micro-structured diffusion plate in micro injection molding were 0.08° and 0.12 μm, respectively. The shape accuracy of the micro-ground mold core and the replication rate of the micro injection molded diffusion plate were 9.2 μm and 90.26%, respectively.
3. The holding pressure has the greatest effect on the light efficiency of the micro-structured diffusion plate. When the melt temperature, injection speed, injection pressure, holding pressure, and holding time were 240 ℃, 40 mm/s, 8.0 MPa, 6.5 MPa, and 2.5 s, respectively, the measured illumination of the micro-structured diffusion plate reached a maximum value of 125.9 Klux, improving the illumination by about 40.82% against the traditional diffusion plate.
4. The prediction accuracies of the designed light efficiency simulation method for the traditional and micro-structured diffusion plates are 94.38% and 90.33%, respectively.

## Figures and Tables

**Table 1:** Simulated illuminances of LED diffusion plates under different V-groove structure parameters.

No.	V-Groove Depth *h* (μm)	V-Groove Angle *θ* (°)	Illuminance *E* (Klux)
1	0	0	94.42
2	100	60	134.29
3	100	90	121.64
4	100	120	103.56
5	100	150	95.49
6	200	60	132.82
7	200	90	123.42
8	200	120	102.55
9	200	150	95.22
10	300	60	138.07
11	300	90	122.04
12	300	120	103.63
13	300	150	94.49
14	400	60	132.79
15	400	90	122.02
16	400	120	102.07
17	400	150	94.45

**Table 2:** The precision grinding conditions of the micro-structured mold core.

CNC Grinding Machine	SMART B818 Ⅲ
Grinding wheel	#600 CBN resin-bonded grinding wheel Wheel speed *N* = 3000 r/min
Workpiece	Super-mirror mold steel (S136H)
Rough grinding	*v*_f_ = 1000 mm/min; *a* = 5 μm, Σ*a* = 290 μm
Fine grinding	*v*_f_ = 1000 mm/min; *a* = 1 μm, Σ*a* = 10 μm
Cooling fluid	Water-soluble coolant
V-groove machining parameters	V-groove angle *α* = 60°, V-groove depth *H* = 300 μm

**Table 3:** Micro injection molding process parameters.

Process Parameters	Level
Melt temperature *T* (℃)	240, 245, 250, 255, 260
Injection speed *V* (mm/s)	40, 44, 48, 52, 56
Injection pressure *P*_i_ (MPa)	8.0, 8.5, 9.0, 9.5, 10.0
Holding pressure *P*_h_ (MPa)	5.0, 5.5, 6.0, 6.5, 7.0
Holding time *T* (s)	1.5, 2.5, 3.5, 4.5, 5.5
